# Miscellany

**Published:** 1903-08

**Authors:** 


### MISCELLANY.

Miss Nellie S. Davis has established a directory of nurses, at the Waldorf Flat No. 1, 1112 Main Street, Buffalo, where nurses may be had at short notice without expense to physicians. The prices range from $7.00 to $25.00 a week, and only nurses of reliability are provided. Applications are received at all times of day or night. Miss Davis refers by permission to Drs. Roswell Park, W. H. Thornton, and N. L. Burnham. Telephone, Bryant 958 R.

